# G-Quadruplexes in Tumor Immune Regulation: Molecular Mechanisms and Therapeutic Prospects in Gastrointestinal Cancers

**DOI:** 10.3390/biomedicines13051057

**Published:** 2025-04-27

**Authors:** Yunxia Zhou, Difei Xu, Ying Zhang, Huaixiang Zhou

**Affiliations:** Tomas Lindahl Nobel Laureate Laboratory, The Seventh Affiliated Hospital, Sun Yat-Sen University, Shenzhen 518107, China

**Keywords:** G-quadruplexes, tumor immune microenvironment, gastrointestinal tumors, treatment strategies

## Abstract

G-quadruplex (G4) is a noncanonical nucleic acid secondary structure self-assembled by guanine-rich sequences. Recent studies have not only revealed the key role of G4 in gene regulation, DNA replication, and telomere maintenance but also showed that it plays a core role in regulating the tumor immune microenvironment. G4 participates in tumor immune escape and the inhibition of immune response by regulating immune checkpoint molecules, cytokine expression, immune cell function, and their interaction network, thus significantly affecting the effect of tumor immunotherapy. This article systematically reviews the molecular mechanism of G4 in tumor immune regulation, especially gastrointestinal tumors, and explores the potential and application prospects of G4-targeted drug strategies in improving anti-tumor immunotherapy.

## 1. Introduction

The discovery and study of G4 structures began in the 1960s, and with a deeper understanding of DNA and RNA molecular structures, G4 has emerged as an important atypical nucleic acid structure that plays a critical role in gene regulation, cell cycle, DNA repair, and telomere maintenance [1,2,3,4,5,6,7,8]. In cancer, neurodegenerative diseases, and viral infections, G4 structures have increasingly become the focus of emerging targeted therapeutic strategies [9,10,11,12]. G4 structures are abundant in proto-oncogene regions of tumor cells, where they are closely related to the transcriptional regulation of oncogenes, tumor immune escape, tumor microenvironment alterations, and therapeutic resistance [9,13,14,15]. Studies have shown that G4 can participate in the immune escape process of tumors by regulating immune checkpoint genes (e.g., PD-L1), inflammatory cytokines, and immune cell functions, thus influencing the effectiveness of immunotherapy [16,17,18]. Furthermore, G4 structures are closely related to DNA replication, genome stability, and telomere maintenance, making them potential targets for anticancer therapy [7,19,20].

As research into G4 structure and function deepens, a growing number of G4-targeted drugs—including G4 stabilizers, unwinders, binders, and G4-based immunotherapy strategies—have been developed [18,21,22,23,24]. These drugs not only show promise in cancer treatment but may also offer novel ideas for treating other diseases. Although the function and mechanism of G4 have been extensively studied in recent years, challenges remain regarding their specific roles in different diseases, the efficacy of targeting strategies, and the clinical translation of these drugs [25,26,27,28]. Particularly in cancer immunotherapy, enhancing the effectiveness of immune checkpoint inhibitors through G4 targeting is a major area of current research [29,30,31,32,33]. This review systematically summarizes the mechanism of action of G4 in tumor immune regulation and gastrointestinal tumors, and explores the therapeutic potential and future application direction of drugs targeting G4, in order to provide theoretical support for cancer treatment strategies.

## 2. Key Features of G-Quadruplexes

### 2.1. Structure of G-Quadruplexes

G4 is a specific nucleic acid secondary structure formed by guanine-rich sequences through Hoogsteen hydrogen bonds [34]. Its basic structural unit, the G-tetrad, is formed by four guanine molecules linked in a planar arrangement, which then stack via π–π interactions to form the quadruplex [35,36]. The stability of G4 structures is influenced by monovalent cations such as K^+^ and Na^+^, with K^+^ providing stronger stabilization due to its optimal ionic radius and coordination properties, as well as its high physiological concentration [37,38]. G4 can adopt various conformations, including parallel, antiparallel, and hybrid forms [36,39,40,41,42] (Table 1).

### 2.2. Distribution of G-Quadruplexes

G4 structures are widely distributed in functional genomic regions:

Telomeres: Telomeric DNA, rich in guanine repeats, readily forms G4 structures that affect telomere maintenance and telomerase activity [7,43].

Promoters: G4 structures are enriched in the promoters of many oncogenes (e.g., MYC, KRAS, BCL-2) and play a role in transcriptional regulation [26,37,44,45,46].

Enhancers and Regulatory Elements: In gene regulatory regions such as super-enhancers, G4 structures can recruit specific proteins to modulate gene expression [47,48,49].

Replication Origins: G4 influences the initiation of DNA replication and collaborates with chromatin remodeling factors [50,51].

Non-coding RNAs: G4 structures have been found in long non-coding RNAs (lncRNAs) and microRNA precursors, affecting RNA processing and function [52,53,54,55,56,57] (Table 1).

### 2.3. Conformational Diversity of G-Quadruplex Structures

Recent advances in high-throughput G4-mapping techniques, such as G4-seq, rG4-seq, and BG4 ChIP-seq, have revealed that G4 structures are not uniformly distributed across the genome and exhibit context-dependent conformational preferences [58,59,60]. While parallel G4 conformations are frequently observed in promoter regions and CpG islands, likely due to their short loop lengths and higher thermodynamic stability, antiparallel and hybrid G4s are more commonly found in telomeres and non-coding regions, reflecting the influence of local sequence context and chromatin structure [2,33]. Moreover, the folding topology of G4s can directly impact their biological functions. For instance, parallel G4s in oncogene promoters (e.g., MYC, KRAS) act as transcriptional repressors by interfering with transcription factor binding or RNA polymerase progression [61], while telomeric hybrid G4s serve as binding platforms for telomere-associated proteins such as POT1 and TRF2, playing critical roles in telomere protection and genome stability [62]. In the 5′-untranslated regions (5′-UTRs) of mRNAs, G4 structures can adopt looped or bulged forms that modulate cap-dependent translation or facilitate ribosome stalling, thereby influencing protein synthesis in a gene-specific manner [63]. Importantly, chromatin environment, supercoiling, and RNA/DNA interactions further modulate G4 folding dynamics and topology in vivo, resulting in functional diversification even among structurally similar motifs [64]. Therefore, G4 structures exhibit conformational diversity across different genomic regions, with their folding topology closely linked to biological functions and jointly regulated by sequence context and chromatin environment.

## 3. Functions of G-Quadruplexes

### 3.1. G-Quadruplexes in Gene Regulation

DNA Replication: G4 can impede DNA replication by causing replication fork stalling. DNA helicases such as BLM and WRN help resolve G4 structures to ensure smooth replication [65].

Transcriptional Regulation: G4 can regulate gene expression by affecting RNA polymerase II binding, obstructing transcription factor binding, or recruiting G4-binding proteins (G4BP) to either activate or repress transcription [66,67,68]. For example, the G4 in the MYC promoter acts as a transcriptional repressor, and small molecule stabilizers like Pyridostatin can downregulate MYC expression [69].

RNA Processing and Translation Regulation: G4 structures in mRNA, particularly in the 5′- and 3′-UTRs, can modulate splicing, translation, and mRNA stability. G4 in NRAS and VEGF mRNAs has been shown to inhibit translation, underscoring its regulatory role in cancer-related genes [70,71,72,73].

Telomere Maintenance: Telomere maintenance is essential for genome stability and cellular lifespan, especially in rapidly dividing tumor cells [74,75]. G4 structures in telomeric regions can inhibit telomerase activity, thereby controlling telomere length and serving as potential targets for anticancer therapy [7,43,76].

**Figure 1:**
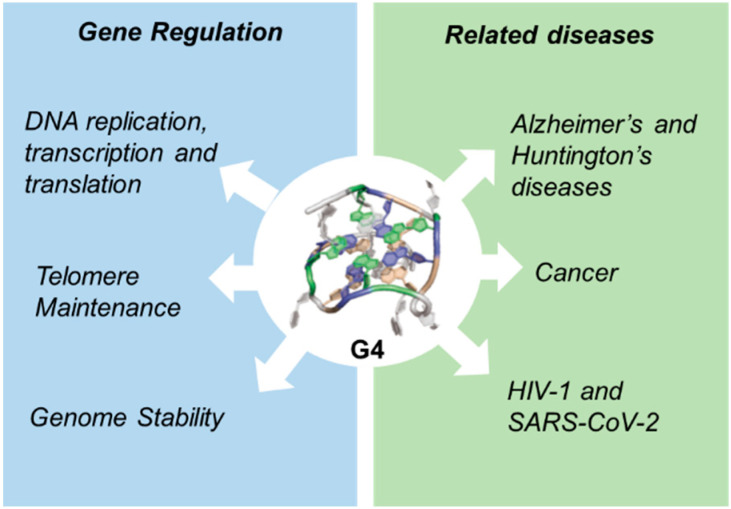
Functions of G-quadruplexes.

Genome Stability: Class switch recombination (CSR) is a biological mechanism by which B cells change the isotype of the antibody they produce (e.g., from IgM to IgG, IgA, or IgE) without altering antigen specificity. This process is crucial for tailoring immune responses and is tightly regulated by activation-induced cytidine deaminase (AID) and associated DNA repair mechanisms [77]. G4 is important for the B-cell lineage and is found in high abundance in Ig variable (V) genes [78] and in so-called “switch” (S) regions targeted by the CSR process [79]. At these locations, G4 plays a regulatory role but can also compromise genomic stability by initiating double-strand breaks (DSBs) and translocations (Figure 1).

### 3.2. G-Quadruplexes in Diseases

Cancer: The presence of G4 in oncogene promoters and telomeres affects oncogene expression, telomere maintenance, and DNA damage responses. Thus, G4 stabilizers (e.g., CX-5461, Pyridostatin) are being explored as novel anticancer agents [16,80,81,82].

Neurodegenerative Diseases: Abnormal G4-mediated RNA regulation has been linked to neurodegenerative disorders such as Alzheimer’s and Huntington’s diseases. For example, G4 structures in the FMR1 gene affect mRNA translation and are associated with Fragile X syndrome [83,84,85].

Viral Infections: G4 plays a role in viral genomes and host antiviral responses. For example, G4 in the HIV-1 and SARS-CoV-2 genomes affects viral RNA translation and replication, suggesting that targeting G4 could be a promising antiviral strategy [86,87,88,89] (Figure 1).

## 4. G-Quadruplexes in Cancer Immunoregulation

Recent studies have found that G4 plays an important regulatory role in the cancer immune microenvironment, mainly mediating tumor cell immune escape and immunosuppression by affecting immune checkpoint molecule expression, cytokine regulation, immune cell function, and molecular interaction networks [4,16,18,30,90] (Table 2).

### 4.1. Transcriptional Regulation of Immune Checkpoint Genes

G-quadruplex (G4) structures have emerged as important epigenetic regulators capable of indirectly modulating PD-L1 transcription, with evidence suggesting context-dependent and sometimes opposing regulatory effects. One study found that certain guanine-rich oligonucleotides, such as nCpG-6-PTO, can form G4 structures and significantly downregulate PD-L1 expression in melanoma cells [100]. However, another study demonstrated that G4 stabilizers, such as CX-5461, can indirectly upregulate PD-L1 expression by activating the cGAS–STING–IFN signaling pathway [16]. Therefore, the regulatory effect of G4 stabilizers on PD-L1 is context-dependent and may involve immune signaling-mediated indirect upregulation. These findings highlight the importance of carefully selecting G4 ligands and optimizing dosing strategies when designing combination therapies with immune checkpoint inhibitors.

### 4.2. Regulation of mRNA Translation and Cytokine Secretion

Beyond their effects on transcription, G4 structures also regulate the translation of cytokines and immune-related molecules through the formation of G4 structures in the 5′-untranslated regions (5′-UTR) of mRNAs [91]. Cytokines such as IL-6, IL-8, and VEGF are critical for immune cell recruitment and activation within the tumor microenvironment [17,101,102]. G4 structures in the 5′-UTRs of these cytokine mRNAs have been shown to influence ribosome binding, thereby modulating translation efficiency [103]. For example, IL-6 and TNF-α exhibit increased translation when G4 structures are disrupted by G4 unwinders, suggesting that G4 stabilization could prevent the upregulation of pro-inflammatory cytokines, thereby influencing immune cell recruitment and activation [104]. The regulation of these cytokines by G4 structures in immune cells has profound implications for immune modulation. Disruption of G4 structures with specific G4 unwinders could therefore alter the inflammatory response and potentially enhance anti-tumor immunity. By modulating cytokine secretion, G4 structures might not only affect the immune landscape but also influence tumor progression, immune resistance, and therapeutic responses.

### 4.3. Immune Cell Function and Tumor Microenvironment

In addition to modulating gene expression at the transcriptional and translational levels, G4 structures also directly influence immune cell function, particularly in T cells, dendritic cells (DCs), and macrophages. These immune cells play pivotal roles in the anti-tumor immune response, and G4 may influence their differentiation, activation, and apoptosis [92,93,94]. For example, in T cells and DCs, G4 may affect their differentiation and activation by regulating DNA damage response (DDR), chromatin remodeling, and epigenetic modifications [95]. Furthermore, G4 structures are involved in the regulation of macrophage polarization. Tumor-associated macrophages (TAMs) often exhibit an immunosuppressive phenotype that supports tumor progression. Targeting G4 structures in these immune cells could potentially shift the macrophage phenotype toward an anti-tumor state, enhancing the overall immune response within the tumor [96]. Further exploration of this mechanism may provide a new theoretical basis for the application of G4-targeted drugs in enhancing anti-tumor immune response.

### 4.4. Molecular Interactions and Epigenetic Regulation

G4 may also work in synergy with epigenetic factors such as non-coding RNA (such as long non-coding RNA, lncRNA) and DNA methylation to further affect gene expression [97,98]. For example, in the regulatory axis (G4–lncRNA–immune gene) in the tumor microenvironment, G4 may act as a signal regulation switch to determine the interaction between tumor cells and immune cells [99]. Therefore, studying how G4 cooperates with these molecular networks to regulate tumor immune responses will help develop more precise targeted intervention strategies.

## 5. Therapeutic Potential of G-Quadruplex Regulation in Gastrointestinal Tumors

As an important secondary structure of gene regulation, G-quadruplex (G4) shows unique biological significance in gastrointestinal diseases, especially gastrointestinal tumors and infectious diseases. Studies in recent years have shown that G4 structure is widely distributed in the promoter region and 5′-UTR regions of oncogenes, and its formation is closely related to the regulation of gene expression [105]. In gastrointestinal stromal tumors (GIST), abnormal expression of the c-KIT gene is a key factor in tumor occurrence and development, and the G4 structure in the c-KIT promoter region has become a potential regulatory target [106,107]. By designing specific small molecules to stabilize the G4 structure, it is not only possible to inhibit the transcription of the c-KIT gene but also to overcome the treatment dilemma caused by kinase inhibitor resistance.

The formation of drug resistance is a major challenge in the treatment of gastrointestinal tumors, and the existence of G4 structure provides a new idea for revealing the mechanism of drug resistance. Studies have found that BCL-2, an anti-apoptotic gene, plays an important role in the drug resistance of GIST cells, and its G4 structure in the 5′-UTR regions may affect the expression level of BCL-2. Certain small molecule G4 binders can specifically target this structure, reduce BCL-2 expression, and thus restore cancer cells’ sensitivity to targeted therapy. In addition, G4 is widely involved in the maintenance of DNA replication and genomic stability [106]. Excessive formation in gastrointestinal tumor cells may lead to blocked DNA replication, thereby affecting the proliferation ability of tumor cells. Therefore, the regulation of G4 structure not only plays an important role in the regulation of oncogene expression but also may become a new strategy to affect the growth of tumor cells.

The impact of G4 structure is not limited to host cells but also plays a key role in pathogens such as Helicobacter pylori. The latest study found that highly conserved G4 structures exist in the nickel transport-related genes (nixA, niuB1, niuB2, and niuD) of the pathogen [108]. These genes regulate the uptake of nickel ions in the host by Helicobacter pylori, and nickel is a key auxiliary factor in maintaining the function of Helicobacter pylori urease and hydrogenase [108]. Therefore, the presence of these G4 structures may affect the survival ability of Helicobacter pylori and provide new targets for anti-infection treatment. Small molecule G4 binders can not only be used to target the G4 structure of oncogenes, but also may stabilize the G4 structure in pathogens, interfere with the expression of their key genes, and ultimately weaken their pathogenicity. This discovery broadens the scope of the application of the G4 structure in disease treatment, making it not only an anti-tumor target but also a new idea for antibacterial therapy.

Targeting the G-quadruplex (G4) structure has become an emerging strategy for the treatment of gastrointestinal tumors. Studies have shown that the G4 structure plays a key role in oncogene expression, drug resistance regulation, and immune escape. Based on this, the development of small molecules or other therapeutic methods that can regulate the G4 structure is expected to provide new treatment options for patients with gastrointestinal tumors.

## 6. G-Quadruplex-Targeted Therapeutic Strategies

The development of therapeutic strategies targeting G4 has gained significant attention in recent years due to their critical role in regulating gene expression and their involvement in cancer progression, immune regulation, and other diseases. G4 structures have become promising targets for both anticancer therapy and immune modulation, with various approaches focusing on stabilizing or unwinding G4 structures to regulate gene expression and enhance immune responses. This section explores the different G4-targeted therapeutic strategies, including G4 stabilizers, G4 unwinders, combination therapies, and multifunctional nanomedicines (Table 3).

### 6.1. G4 Stabilizers

G4 stabilizers are small molecules that enhance the formation and stability of G4 structures, particularly in promoter regions of oncogenes, immune checkpoint genes, and other critical regulatory sites [16,58]. By stabilizing G4 structures, these agents can inhibit the transcription of target genes, suppress tumor growth, and enhance the immune response [18,118]. Several G4 stabilizers, such as CX-5461 (Pidnarulex), Pyridostatin, BRACO-19, and TMPyP4, have been identified and shown to selectively bind to G4 structures, blocking the activity of transcription factors or RNA polymerase [109,110,111]. The FDA has granted Fast Track designation to CX-5461 as a potential treatment option for breast and ovarian cancer patients harboring mutations in BRCA1/2, PALB2, or other homologous recombination deficiencies [119]. A G4-stabilizing compound, CX-5461 is designed to stabilize the folded conformation and synergize with homologous recombination (HR) repair pathway defects; this prevents DNA breaks at replication forks and leads to cancer death [120]. Stabilization of G4 at replication forks can lead to significant genomic instability and DNA breakage.

### 6.2. G4 Unwinders

G4 destabilizers, also known as G4 destabilizers, work by promoting the dissociation of G4 structures to prevent replication fork stalling and DNA breakage. For example, Pif1 DNA helicase and 2′-F cytidine trimers (2′-F C3). G4 structures are formed in vivo, and they are resolved by Pif1 DNA helicase [112]. 2′-F C3 can release the translation of mRNA containing G-quadruplexes without inducing DNA damage [24]. In the context of immunotherapy, G4 unwinders can be used to modify the expression of cytokines, immune checkpoint genes, and other immune-regulatory factors, thereby enhancing anti-tumor immune responses. The use of G4 unwinders in combination with immune checkpoint inhibitors may enhance the efficacy of immunotherapies by promoting immune cell activation and cytokine production.

### 6.3. Combination Therapies

Given the complex role of G4 in regulating immune checkpoint genes and tumor growth, combination therapy targeting G4 structures combined with traditional immunotherapy and chemotherapy drugs has significant prospects. G4-targeted drugs can be used in combination with immune checkpoint inhibitors (such as anti-PD-1/PD-L1 antibodies) to form a double-hit strategy, that is, reducing PD-L1 expression through G4 regulation and enhancing T cell function through PD-1 inhibitors to improve the therapeutic effect [16]. Chemotherapeutic drugs that induce DNA damage (such as cisplatin) can synergize with G4-targeted therapy to further induce genomic instability in tumor cells, thereby enhancing the overall therapeutic effect [113]. In addition, G4 regulation may also enhance the effect of tumor vaccines or CAR-T therapy, but the current dose matching, treatment window, and long-term safety still need further exploration [114].

### 6.4. Multifunctional Nanomedicines

Nanotechnology has shown great potential in G4-targeted therapy. For example, nanocarriers for targeted delivery of G4 stabilizers can improve drug selectivity, stability, and bioavailability while reducing systemic toxicity [115,116]. In addition, nanomaterials can also be used to co-deliver G4-targeted drugs with immunotherapeutic molecules, such as nanoencapsulated anti-PD-L1 antibodies combined with G4 modulators to enhance anti-tumor immune responses. Multifunctional nanomedicines that combine G4 modulators with immune checkpoint inhibitors, chemotherapeutic drugs, or cytokines have the potential to enhance the therapeutic effects of cancer immunotherapy [117]. For example, nanoparticles encapsulating G4 stabilizers can be designed to selectively release drugs at tumor sites, improve pharmacokinetics, and minimize off-target effects These nanocarriers can also be used to co-deliver anti-PD-L1 antibodies to enhance anti-tumor immune responses while modulating G4-related immune pathways.

## 7. Conclusions and Outlook

In summary, G4 structures are emerging as critical regulatory elements that influence gene transcription, DNA replication, telomere maintenance, and RNA metabolism. Recent evidence underscores their pivotal role in modulating the tumor immune microenvironment by affecting immune checkpoint expression, cytokine production, immune cell function, and various molecular interaction networks. These multifaceted functions position G4 as a promising target for innovative cancer immunotherapeutic strategies.

Despite the rapid advances in G-quadruplex (G4)-targeted cancer therapy, several critical challenges must be addressed before clinical translation can be achieved. G4-stabilizing ligands, such as CX-3543 (Quarfloxin) and CX-5461, have shown promising antitumor activity by stabilizing G4 structures at oncogene promoters or rDNA regions, leading to transcriptional repression and DNA damage [16,121,122,123]. However, both compounds exhibit off-target effects and limited pharmacokinetic profiles; notably, CX-3543 failed in clinical trials due to poor bioavailability and high plasma protein binding [123]. Moreover, the dynamic nature of G4 structures across different genomic contexts complicates their selective targeting in cancer versus normal tissues [124]. Targeting G4-unwinding helicases, such as WRN, has gained attention, especially in microsatellite instability-high (MSI-H) tumors where WRN is synthetically essential [125]. WRN inhibitors like NSC 617145 and ML216 sensitize cells to replication stress, yet their systemic inhibition may also impair genome stability in normal proliferating cells [126], raising concerns of potential toxicity. Combination therapies present a rational strategy to enhance the efficacy of G4-targeted agents. For instance, CX-5461 synergizes with PARP inhibitors in BRCA-deficient tumors by exacerbating replication stress [127], and G4 ligands have also been shown to boost immune checkpoint blockade by enhancing tumor immunogenicity type I interferon responses [4,18].

Nevertheless, combination regimens must be cautiously optimized to avoid overlapping toxicities and unintended immunosuppressive effects. To improve tumor-specific delivery and reduce systemic exposure, various nanoparticle-based systems have been developed to encapsulate G4 ligands. For example, liposomal formulations of GQC-05, a G4-stabilizing ligand, improved its solubility and tumor targeting in preclinical leukemia models [128]. Similarly, AS1411, a G-quadruplex-forming aptamer, was utilized as a tumor-targeted delivery vehicle for TMPyP4. The AS1411–TMPyP4 complex exhibited enhanced cellular uptake and accumulation in tumor cells, leading to improved photodynamic therapeutic efficacy [129]. However, challenges such as immune clearance, off-target uptake, tumor heterogeneity, and large-scale production still limit the clinical application of these systems. An additional concern is the emergence of resistance mechanisms. Tumors may adapt to G4 stress by upregulating G4-resolving helicases (e.g., BLM, PIF1), mutating G4-forming regions, or reprogramming DNA repair pathways [130,131]. To overcome such resistance, researchers are exploring dual-function compounds that simultaneously target G4s and helicases, or combining G4 ligands with immune modulators to reinforce therapeutic pressure. Importantly, the lack of robust in vivo models that faithfully recapitulate the immunological and transcriptional consequences of G4-targeting remains a major limitation. The development of G4-reporter mouse models, immune-competent tumor-bearing mice, and spatial transcriptomic tools to visualize G4 dynamics will be essential for future mechanistic and therapeutic validation.

In summary, while G4-targeted strategies hold immense promise for tumor immunomodulation, their translation requires solving multiple pharmacological, biological, and immunological challenges. Addressing these issues through innovative drug design, biomarker-driven patient selection, and mechanistically guided combination regimens will be crucial for unlocking the full potential of G4 biology in cancer therapy.

Looking ahead, the integration of advanced multi-omics analyses with nanotechnology is expected to accelerate the identification of novel G4 biomarkers and therapeutic targets. This interdisciplinary approach could facilitate the development of personalized cancer immunotherapies that leverage the unique regulatory capabilities of G4 structures. Future research should prioritize the refinement of G4-targeting agents for enhanced specificity and minimal side effects, as well as the design of rigorous clinical trials to evaluate their safety and efficacy.

Ultimately, continued efforts to understand and manipulate G4-mediated regulatory networks hold promise for overcoming current limitations in cancer immunotherapy and may establish G4-targeted strategies as a cornerstone of precision oncology.

## Figures and Tables

**Table 1:** The key characteristics of quadruplex.

Features	Description
Basic structure	G-tetrad is formed by Hoogsteen hydrogen bonding
Stability Factors	Stabilized by monovalent cations (K^+^, Na^+^); magnesium ions can further enhance stability
Structural Types	Parallel, antiparallel, and hybrid; can form single-stranded, double-stranded, or multi-stranded structures
Genomic Localization	Located in telomeres, promoters, enhancers, replication origins, and non-coding RNA regions

**Table 2:** Mechanisms of G-quadruplexes in cancer immune regulation.

Mechanism	Molecular Targets	Regulation	Biological Effects	Ref.
Immune Checkpoint Gene Regulation	PD-L1	G4 promotes transcription	Reduces immune suppressive molecule expression, enhances T cell cytotoxicity	[16]
mRNA Stability and Translation Regulation	IL-6, IL-8, IFN-γ, TNF-α	G4 enhances or inhibits mRNA stability	Affects cytokine secretion, regulates inflammation and immune microenvironment	[91]
Immune Cell Function Regulation	T cells, DCs, macrophages	G4 regulates DNA damage response and epigenetic modifications	Influences immune cell differentiation, activation, and antigen presentation capacity	[92,93,94,95,96]
Molecular Interaction Network Influence	lncRNA, DNA methylation	G4 mediates gene regulatory networks	Affects the epigenetic regulation of immune genes, shapes tumor microenvironment features	[97,98,99]

**Table 3:** G-quadruplex-targeted therapeutic strategies.

Strategy	Representative Drugs	Mechanism of Action	Potential Advantages	Challenges and Limitations	Ref
G4 Stabilizers	BRACO-19, TMPyP4, CX-5461, Pyridostatin	Stabilize G4 structures and inhibit target gene transcription (e.g., c-MYC, PD-L1)	Reduce tumor immune escape, enhance T cell cytotoxicity	Low selectivity, may affect normal cell gene expression	[109,110,111]
G4 Unwinders	Pif1 DNA helicase, 2′-F C3	Promoting the dissociation of G4 structures to prevent replication fork stalling and DNA breakage	Without Inducing DNA Damage	Potential for drug resistance or non-specific effects	[24,112]
Combination Immunotherapy	G4 Stabilizer + PD-1 Antibody	Modulate G4 to reduce PD-L1 while blocking the PD-1/PD-L1 pathway	Enhance the efficacy of immune checkpoint inhibitors	Requires optimization of dose matching and immune tolerance risks	[16,113,114]
Nanomedicine Delivery	G4-targeted Nanocarriers	Deliver G4 modulators to improve drug selectivity and stability	Reduce systemic toxicity, improve tumor targeting	Delivery system still needs optimization, significant clinical translation challenges	[115,116,117]
